# Optimizing strain selection for association studies under hard cost constraints

**DOI:** 10.1093/genetics/iyag137

**Published:** 2026-05-27

**Authors:** Christoph D Rau, Patrick H Bradley

**Affiliations:** Department of Genetics and Computational Medicine Program, University of North Carolina at Chapel Hill, Chapel Hill, NC 27599, United States; Department of Microbiology, The Ohio State University, Columbus, OH 43210, United States

**Keywords:** optimization, strain selection, GWAS, animal models, phylogenetic regression

## Abstract

Quantitative genetics methods linking genotype to phenotype are powerful tools in model organisms and nonhuman populations, allowing researchers to apply well-controlled perturbations to commercially available strain collections. However, purchasing and phenotyping large collections can be cost-prohibitive, and it is unclear how to select smaller subsets to maximize statistical power for a given budget. We evaluated several strain selection methods considering cost, genetic diversity, or both, using simulations from 2 settings—phylogenetic regression across bacterial isolates and linear mixed-model regression across mouse strains—as well as real subsampled data on cardiovascular phenotypes from the Hybrid Mouse Diversity Panel. Surprisingly, considering only costs while ignoring genetic diversity (MinCost) was typically one of the most powerful approaches. Considering diversity alone tended to yield low power, likely because it resulted in fewer total strains being selected. Methods weighting both cost and diversity could sometimes outperform MinCost, but this depended on the scenario and how diversity was maximized. Counterintuitively, the most commonly studied diversity-maximizing objective—maximizing the minimum pairwise distance between selected strains (MaxMin)—had the lowest power. The most powerful objectives included 2 not previously applied for this purpose: maximizing the total sum of pairwise genetic distances (MaxSum) and maximizing total minor allele frequency (MaxMAF). A new cost-aware p-Median approach, which selects the most representative strain subset, also performed well, especially on real data. Overall, when strains have unequal costs, maximizing sample size under a given budget should typically take priority over maximizing genetic diversity, though some methods co-optimizing both may retain more power.

## Graphical Abstract

**Graphical Abstract:**
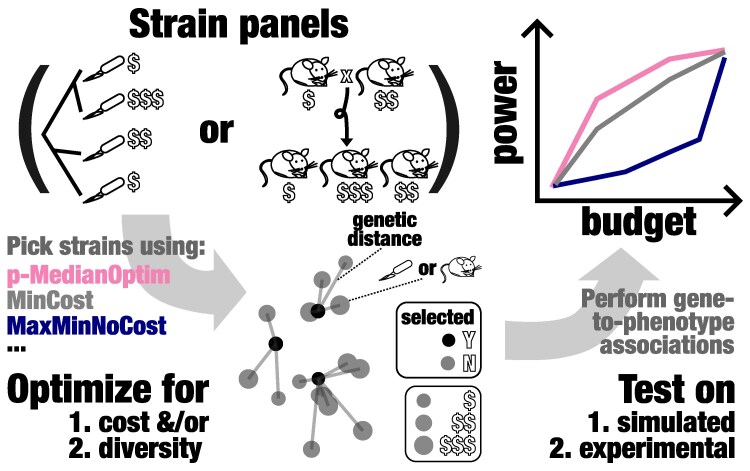

## Introduction

The cost of research has grown significantly over the past 2 decades. One measure is the average size of an R01 grant from the National Institutes of Health, which has increased 87% between 1999 and 2019 (National Institutes of Health 2024), 64% faster than inflation. These cost increases come from many factors: novel techniques and technologies that supplant other, often cheaper, approaches (e.g. PCR to RNA microarrays to RNAseq to single cell RNAseq), more complex study designs which necessitate additional research cohorts and endpoints, and, notably, a drive to increase the size of studies to attempt to both achieve greater statistical power and identify less penetrant drivers of disease conditions (Wright and Levine 2003; Gajović and Pochet 2016; LeBel et al. 2017). For example, over the past 2 decades, the GWAS community has been gathering larger and larger populations to try and recover the effects of polymorphisms with smaller and smaller effect sizes (Yengo et al. 2022; Abdellaoui et al. 2023). Depending on how they are funded, such large populations may be out of reach for individual investigators. Furthermore, beyond simple financial considerations, researchers who use animal populations are often mandated to follow the 3 Rs (replacement, reduction, refinement) in their research, which states that if it is possible to achieve the same or similar result with fewer animals, then researchers should make every effort to do so; for example, the US Food and Drug administration has recently announced a roadmap to reduce animal testing in preclinical studies (2025). Taken together, the increasing costs in both money and time of doing research coupled with ethical responsibilities to animal cohorts are powerful incentives to optimize and streamline research projects, as long as this does not come at the expense of statistical power.

In comparative studies, one opportunity for streamlining concerns how we select the strains that will be phenotyped. Diverse collections of microbes have proven to be powerful tools for finding genetic variants associated with phenotypes including host range of pathogens (Sheppard et al. 2013; Tiwari et al. 2023), positive fitness effects of symbionts (Batstone et al. 2022), toxin tolerance in industrially relevant microbes (Sardi et al. 2018), and gene and protein expression in model organisms (Teyssonniere et al. 2024). When applied in diverse animal lines, GWAS, MPRA assays, and other comparative omics-scale analyses have allowed us to link genetic polymorphisms to complex disease susceptibility (Rau et al. 2015a, 2015b, 2017; Salimova et al. 2019; de Conti et al. 2020; Chella Krishnan et al. 2023). However, prices for such strains are not uniform: rather, they vary widely across strain collections, individual genotypes, and even how a particular strain is propagated and shipped. For example, a mouse from Jackson Labs costs a different amount of money depending on its strain, age, and sex, ranging from less than $30 for a 3-wk-old C57BL/6J male, to over $260 for a 9-wk-old NZO/HILtJ male, to nearly $2,500 a litter for one of the many cryopreserved strains available for purchase. Similar factors apply to bacterial strains. Type strains are required to be preserved in at least 2 repositories, which may have different costs: the type strain of *Lachnospira eligens*, shipped as lyophilized cells, costs $474 from the American Type Culture Collection (ATCC) but 100 EUR (∼$113) from the German Collection of Microorganisms and Cell Cultures (DSMZ). As with mice, the format also matters: a live, active culture of the *L. eligens* type strain from DSMZ would cost 240 EUR ($273). Finally, both ATCC and DSMZ, as well as many other repositories, have unique strains that can only be purchased from them. Being able to optimize the selection of strains to maximize statistical power for a given budget would be a major benefit for the labs which use these populations.

Several families of approaches for selecting representative subsets from larger cohorts have been developed, largely in the contexts of bioconservation and genotype imputation. These include methods that maximize phylogenetic diversity (MaxPD methods) (Faith 1992; Pardi and Goldman 2005, 2007; Steel 2005; Hartmann and Steel 2006; Kang and Marjoram 2012; Zhang et al. 2013), maximizing the minimum pairwise distance between selected taxa (MaxMin methods, equivalent to the p-dispersion problem under no-cost constraints) (Kuby 1987; Hartmann and Steel 2006; Bordewich et al. 2008) and minimizing the average distance from non-selected samples and selected samples (p-median methods) (Kaufman and Rousseuw 1990a; Faith 1994; Matsen et al. 2013; Kang et al. 2015). Most of these methods, however, were developed in the context of equal-costs, where each sample is equally weighted, with relatively few studies testing how they perform under realistic, unequal cost constraints, even though Weitzman's original formulation of the “Noah's Ark” problem explicitly called for incorporating costs (Weitzman 1998). Furthermore, maximizing genetic diversity may not translate into conserving statistical power (McAuliffe et al. 2005), and many approaches which rely on phylogenetic trees cannot capture genetic relationships shaped by horizontal gene transfer or complex kinship structures from backcrosses (Smillie et al. 2011). Beyond these established approaches, additional metrics which have received less attention in genetics, such as maximizing the total pairwise genetic distance (MaxSum approaches) (Kuby 1987; Martí et al. 2022) and maximizing the sum of minor allele frequencies (MaxMAF approaches, similar to the “M Strategy” from crop science literature) (Schoen and Brown 1993; Kim et al. 2007), could potentially offer better performance but have not been systematically evaluated.

**Fig. 1.**
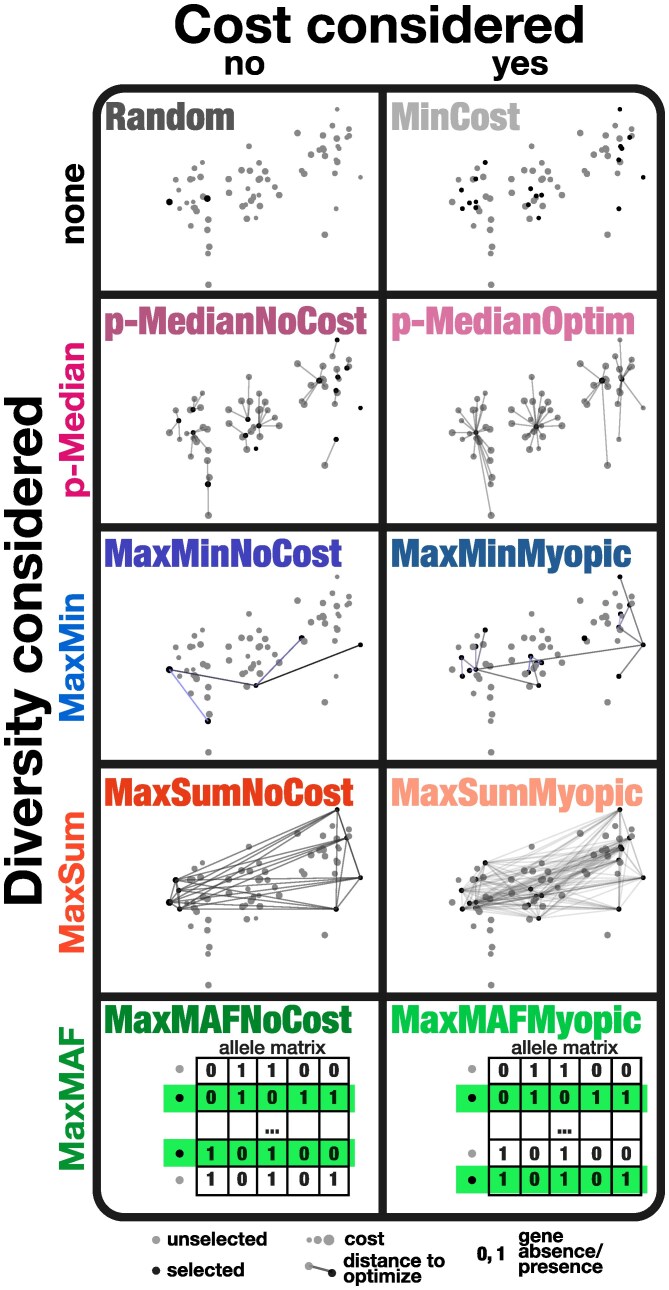
Illustration of methods considered for strain picking. Each method is illustrated with an example where selected strains are represented by black circles and unselected strains by gray circles, with cost being represented by the circle size. Methods in the same column either do not (left) or do (right) consider the cost of strains, while methods in the same row either do not consider diversity (top, Random and MinCost) or consider it in the same way (other rows). The p-Median, MaxMin, and MaxSum methods all optimize a function of distances between strains (gray lines connecting circles). The p-Median methods minimize the total distance between unselected strains and the nearest selected strain. The MaxMin methods maximize the minimum distance between selected strains, while the MaxSum methods instead maximize the total distance. Instead of distances, the MaxMAF methods maximize the sum of MAFs, shown by the inset matrices. “Myopic” indicates a greedy search that prioritizes strains by diversity divided by cost, while “Optim” indicates a method that directly optimizes the objective using ILP. The “NoCost” methods also use greedy search, except for “p-MedianNoCost” which runs the Partitioning Around k-Medoids algorithm with successively greater *k*.

In this manuscript, we set out to study different approaches for strain selection under a hard cost constraint and to quantify how these methods affect our power to detect genetic associations (Fig. 1). Because we wanted to apply these methods in settings where a tree might not best describe the genetic relationships between strains, we did not further consider methods for maximizing PD and instead focused on approaches that allowed more flexible measures of genetic distance. Here, we consider 5 families of methods for considering genetic diversity:

- None: Only the number of strains is considered.
- p-Median: Minimize the total minimum genetic distance between non-selected and selected strains (as with the min-ADCL method).
- MaxMin: Maximize the minimum genetic distance between selected strains.
- MaxSum: Maximize the total genetic distance between all pairs of selected strains.
- MaxMAF: Maximize the sum of minor allele frequencies (MAFs).

For each family, we test an algorithm that does not consider cost (“NoCost”), simply stopping when the budget is exhausted and at least one algorithm that does (denoted by either “Myopic” or “Optim”). The “NoCost” algorithm is a greedy search, taking the strain that improves the family objective the most at each step, except in the case of “p-MedianNoCost,” where greedy search is known to perform poorly and we instead use the Partitioning Around Medoids algorithm. The “Myopic” algorithms we consider are greedy searches that divide a diversity measure by the cost of the strain, as with the “myopic rule.” Finally, the “Optim” algorithm directly optimizes the objective via mixed-integer linear programming or MILP.

To our knowledge, this is the first systematic test of how different methods for cost-constrained strain selection affect power in downstream gene association studies. Additionally, the few studies to consider costs have focused on the MaxPD and MaxMin objectives; we have not found any studies that have implemented and tested cost-aware versions of the alternative MaxSum, MaxMAF, or p-Median approaches.

We evaluate these methods using simulations, which we conduct in 2 different settings: phylogenetic regression in microbes and GWAS in mice. In the first setting, we model the effects of strain selection on power in phylogenetic regression applied to synthetic data from genotyped bacterial isolates. We perform 2 tests, one where we use the real genotypes of isolates from Lachnospiraceae, the most prevalent gut bacterial family worldwide, and one where we introduce a form of study bias by simulating an additional oversampled clade. In the second setting, we model the effects of strain selection on GWAS results in the Hybrid Mouse Diversity Panel (HMDP), a commonly used genetic reference population for studying a number of phenotypes and diseases (Ghazalpour et al. 2012; Davis et al. 2013; Lusis et al. 2016; Cao et al. 2022; Lahue et al. 2025). In our GWAS simulations, we vary the MAF and effect size (χ^2^) of causal SNPs and also explore the impact of reducing the variance of prices by adding extra equal, fixed costs per strain (as might be appropriate when the cost of strain maintenance and/or phenotyping is a non-negligible portion of a research plan's budgetary constraints). Finally, we follow up our simulation studies with an analysis of actual data from an HMDP GWAS study in which the beta adrenergic agonist isoproterenol was used to induce cardiac hypertrophy, which we subsample to simulate subset selection.

## Methods

### Data sources

Mouse genotype data from the Mouse Diversity Array for the HMDP was obtained from the Mouse Genomics Informatics website (Rau et al. 2015a). Mouse phenotype data is from a previously published dataset (Rau et al. 2015b) and is available at Mendeley Resources at data.mendeley.com/datasets/y8tdm4s7nh/1 (Rau 2016). Mouse costs were pulled from the Jackson Labs website (Labs 2024) as on 4/05/2024 and represent the cost of 1 male and 2 female mice or 1 litter of cryopreserved animals, depending on the current status of the animals at the time of accession.

Metadata for bacterial strains was downloaded from BacDive (Schober et al. 2025) as of 07/31/2025. Strains with a genome in NCBI's GenBank or RefSeq were retained, then matched with the metadata from the Genome Taxonomy Database (GTDB) (Parks et al. 2022) release 220 (downloaded 10/31/2024) to obtain taxonomic assignments, ignoring genome version numbers. Genomes were then filtered for GTDB annotations for the family Lachnospiraceae. The 5 most common strain repositories for these genomes were the German Collection of Microorganisms and Cell Cultures (DSMZ), Japan Collection of Microorganisms, ATCC, Culture Collection University of Gothenburg, and Korean Collection for Type Cultures (KCTC). Costs for each repository were estimated by spot-checking 5 of the Lachnospiraceae strains on the respective websites and averaging the results; shipping costs were not included. When the exact same strain was available in multiple collections, we retained the cheapest copy. We used OrthoFinder (Emms and Kelly 2019) to identify orthogroups (i.e. genes descending from a common ancestor in this group of species). Orthogroups with identical distributions across genomes were merged into phylogroups and then filtered such that all remaining phylogroups were present in at least 3 genomes and absent in at least 3 genomes.

### Simulation studies

#### Phylogenetic regression

We performed 2 sets of tests: one using real Lachnospiraceae genotypes and the real tree (“Real”) and one using simulated genotype data on a version of the real Lachnospiraceae tree that had been augmented by a set of 100 very similar strains with very low cost (“Augmented”), to mimic a situation where one taxon may be highly oversampled relative to all others.

#### Real

We started with the Lachnospiraceae genome tree generated by OrthoFinder, making it ultrametric by using relative evolutionary divergence normalization (Parks et al. 2018) using the “castor” package. For each scenario, we simulated 5,000 phenotypes on this ultrametric genome tree under a Brownian motion model using the “rTrait” function in the “phylolm” package. For each phenotype, we picked a phylogroup (without replacement) and then added an offset to that phenotype when the phylogroup was present, with the offset corresponding to 60% of that phenotype's standard deviation. Phylogroups present in fewer than 3 genomes or absent in fewer than 3 genomes were excluded. We finally performed one phylogenetic regression per phenotype and phylogroup pair, allowing for measurement error, using the phylolm function in the “phylolm” package. We estimated power as the proportion of these tests with a *P*-value less than 0.05, replacing any tests that returned an NA value (as might happen when, e.g. there is no variance in the phylogroup) with a *P*-value of 1. We estimated false positives in the same way but using phenotypes where the phylogroup offset was not added.

#### Augmented

Starting with the above tree of Lachnospiraceae genomes, we first grafted a random coalescent tree of 100 strains onto the immediate ancestor of *Agathobacter rectalis*, with the random tree scaled to 1/10th the node depth of this ancestor. The presence/absence vectors of 5,000 synthetic phylogroups were then simulated using the rBinTrait function in phylolm (Ho and Ané 2014), which uses a continuous time Markov chain approach to model binary traits along a tree. For each synthetic phylogroup, the parameters alpha and beta were obtained by performing Ives-Garland phylogenetic logistic regression (Ives and Garland 2010) on one of the 5,000 phylogroups selected in the “real” simulation, using the phyloglm function in the phylolm package (Ives and Garland 2010). We used rejection sampling to make sure that all simulated phylogroups were present in at least 3 genomes and absent in at least 3 genomes. Finally, phenotypes were simulated and power and false positives were assessed as in the “real” simulation.

#### Recombinant

To assess the impact of genetic structure on the results, we first selected a set of 6 “parental” Lachnospiraceae strains using PAM (Kaufman and Rousseuw 1990b) and then added a set of 100 simulated strains where alleles were sampled with equal probability from 1 of the 6 parents, and costs were sampled from a discrete uniform distribution, ranging from $20 to $200 in steps of $10. Strain picking, phenotype simulation, and power and false-positive assessment were all performed as above.

#### GWAS phenotype creation

We created sets of 100 simulated phenotypes each for our simulation studies. For each simulated phenotype, we drew a SNP at random that matched a required MAF criterion (5% to 10%, 10% to 30%, or 40% to 50%). We then selected a β value for this SNP based on a desired χ^2^ threshold (10, 50, or 100) and added noise based on a multivariate normal (MVN) derived from 2 variance terms, the first the proportion of variance attributable to genetic effects ($\sigma_{g}^{2}$), set at 40% consistent with our past work (Rau et al. 2020), and the second the proportion attributable to all other sources of variation ($\sigma_{e}^{2}$) as follows:


$$y=\beta X+MVN(0,\sigma_{g}^{2}K+\sigma_{e}^{2}I)$$

where **K** is the kinship matrix of the strains of the HMDP calculated using the kinship function of the myFastLMM R package (Lippert et al. 2011).

#### GWAS approach

Simulated phenotype–genotype pairs were analyzed using the linear mixed-model approach fastLMM (Lippert et al. 2011). Power was calculated based on the number of associations that met a genome-wide significance threshold of 4.2E−6 as previously determined in the HMDP (Bennett et al. 2010; Ghazalpour et al. 2012; Rau et al. 2015a; Lusis et al. 2016). At a χ^2^ of 100, there was 100% power in the full panel (Supplementary Fig. 1a). In the χ^2^ of 50 set, power ranged from 82% to 93% depending on the MAF (Supplementary Fig. 1a). Power in the χ^2^ of 10 set of simulated phenotypes was very low (<10%) in the whole panel and was not used going forward.

### Strain subset selection

We selected strain subsets for our simulation study using the following algorithms.

#### Greedy approaches

##### Random

Strains were selected randomly until the budget was exhausted.

##### MinCost

Strains were ranked from cheapest to most expensive, breaking ties randomly, then selected in that order until the budget was exhausted.

##### MaxMinNoCost

Strains were selected according to a greedy algorithm that chooses the most diverged strain at every step, regardless of cost. This is represented by the following pseudocode, where *n* is the total number of strains, *x* is a binary vector with *x_i_* representing whether strain *i* is selected, c*_i_* is the cost of strain *i*, *B* is the total budget, and *d_i,j_* is the genetic distance between strains *i* and j:

1) cheapest = which(c == min(c))
2) x[sample(cheapest, 1)] = 1 # *random initialization*
3) while (B > 0):
  a) candidates = which(x == 0 & c <= B)
  b) selected = which(x == 1)
  c) for (a in candidates):
    i) mindist_a = min(d_{a,s}) for s in selected)
  d) new = which(mindist_a == max(mindist_a))[1]
  e) x[new]=1
  f) B = B – c[new]

##### MaxMinMyopic

This method uses the same algorithm as MaxMinNoCost, except that the minimum distances are scaled by cost, meaning that step 3d would read:

- new = which(mindist_a/c_a == max(mindist_a/c_a))[1]

##### MaxSumNoCost

This method uses a similar approach to MaxMinNoCost, but steps 3c-d are different:

1) cheapest = which(c == min(c))
2) x[sample(cheapest, 1)] = 1
3) while (B > 0):
  a) candidates = which(x == 0 & c <= B)
  b) selected = which(x == 1)
  c) for (a in candidates):
    i) sumdist_a = sum(d_x, y for x, y in union(a, selected))
  d) new = which(sumdist_a == max(sumdist_a))[1]
  e) x[new] = 1
  f) B = B – c[new]

##### MaxSumMyopic

As MaxSumNoCost, but as before, the sum of distances is scaled by cost in step 3d:

- new = which(sumdist_a/c_a == max(sumdist_a/c_a))[1]

##### MaxMAFNoCost

This method uses a similar framework as the algorithms above, where we choose the next strain such that it minimizes the following equation:


$$\overset{m}{\underset{i=1}{\sum }}\;\left(0.5-\left|\left(\frac{\sum_{\;j=1}^{n}\;x_{i,j}}{\left\|x_{i}\right\|}\right)-0.5\right|\right)$$

Here, *x* is a *m* × *n* matrix in which there are m SNPs; the first (*n*–1) columns are the currently selected strains, and the nth column is the “applicant” strain. This algorithm acts to select strains such that they push the MAF of the selected strains as close to 50% as possible, thereby attaining “maximum MAF.” In pseudocode:

1)cheapest = which(c == min(c))
2)x[sample(cheapest, 1)] = 1
3)while (B > 0):
  a) candidates = which(x == 0 & c <B)
  b) selected = which(x == 1)
  c) for (a in candidates):
    i) sumMAFs_a = sum(0.5 – abs(rowMeans(snp_matrix[, union(selected, a)]) – 0.5))
  d) new = which(sumMAFs_a == max(sum_MAFs_a))[1]
  e) x[new] = 1
  f) B = B – c[new]

##### MaxMAFMyopic

As MaxMafNoCost, but as before, sumMAFs_a is replaced by sumMAFs_a/c_a in step 3d:

- new = which(mindist_a/c_a == max(mindist_a/c_a))[1]

#### Other heuristic approaches

##### p-MedianNoCost

Because greedy search does not work well for the p-Median problem, we instead used the fast Partitioning Around (k-)Medoids method (Kaufman and Rousseuw 1990b) implemented in the pam function in the “cluster” package in R (Maechler et al. 2025). The PAM algorithm for p-Median problems involves making iterative improvements to the set of selected medoids, but unlike a pure greedy search, it allows medoids to be replaced. To handle the cost constraint, we ran PAM with k increasing from 2 to *n*-1, where *n* is the total number of strains. We kept solutions that were under budget and of these kept the result that selected the most strains.

#### Optimization-based approaches

##### p-MedianOptim

We formulated this problem to be similar to minimum-ADCL (Matsen et al. 2013) and k-medoids, with an additional constraint on total cost and with no predetermined number k of representatives. Thus, we were interested in picking k-medoids or representative strains with minimum distance to the closest nonchosen strains for each representative, with k allowed to vary between one and the number of strains.

The p-MedianOptim method then minimizes the following sum:


$$\overset{n}{\underset{\;j=1}{\sum }}\;\overset{n}{\underset{i=1}{\sum }}\;d_{i,j}y_{i,j},\;i,j\in \{1..n\}$$

subject to the following constraints:

1.
$\overset{n}{\underset{i=1}{\sum }}\;y_{i,j}=1,\;\;j\in \{1..n\}$

2.
$0\leq x_{i}-y_{i,j}\leq 1,\;\;i,j\in \{1..n\}$

3.
$x_{i},\;y_{i,j}\in \{0,\;1\},\;\;i,\;j\in \{1..n\}$

4.
$\overset{n}{\underset{i=1}{\sum }}\;x_{i}c_{i}\leq c_{tot},\;\;i\in \{1..n\}$

5.
$0\leq \overset{n}{\underset{i=1}{\sum }}\;x_{i}\leq n,\;\;i\in \{1..n\}$

The first constraint guarantees that exactly one “nearest” representative is assigned to each of full set of strains. The second guarantees that $y_{i,j}$ is never 1 when $x_{i}\;$ is not also 1: this in turn means that only distances from a representative to its assigned strains will be summed in the objective function. The third guarantees that *x* and *y* are binary, the fourth gives the cost constraint, and the fifth allows for any number of strains *k*  $\left(k=\sum x_{i}\right)$ to be selected. Strains that must be selected (for example, strains that are already part of a researcher's collection) can also be included as additional constraints on the value of a particular $x_{i}$.

The method was implemented in R, using the “highs” package (Schwendinger et al. 2025) which provides bindings to the open-source HiGHS solver (Huangfu and Hall 2017).

We did not evaluate an integer linear programming (ILP) version of the MaxSum or MaxMin problems because even in tests on very small datasets, their performance was extremely slow (see Discussion). We also did not attempt to frame MaxMAF as an ILP problem because it uses the entire allele frequency matrix instead of a distance matrix and would therefore require very large models with orders of magnitude more variables than the other ILP methods tested here.

### Fitting and significance testing

We fit generalized additive models (GAMs) to the power estimates, assuming the data were beta-distributed, using the restricted maximum-likelihood estimator the R package “mgcv” (Wood 2010). Because all methods eventually converge given a high enough budget, for the purpose of the model fit, we kept only budget values where the average power across every method was less than 90% of the maximum power observed. To reduce the impact of exact zeros at the low end, which distort the fit, we also slightly moderated power values by adding 0.005 and divided by 1.005. Power was then modeled as a per-method smooth function of the budget, using k = 5 knots. After fitting GAMs, the estimated marginal means over these budget ranges were calculated using the “emmeans” R package. Using the “multcomp” (Hothorn et al. 2008) package, significant pairwise differences at *P* = 0.05 were then assessed using the Tukey adjustment, from which compact letter displays were finally generated.

### Validation approaches

*HMDP:* The right ventricular weight phenotype after isoproterenol stimulation was selected as it had the largest number of genome-wide significant loci from its manuscript (Fig. 6a) (Rau et al. 2015b). The mice used in the selected study did not comprise the entire panel but instead consisted of 93 strains, which we calculated to be a total cost of $119,241. Costs were set at $20,000 intervals from $0 to $100,000 and all selection algorithms were run 10 times per approach. Included SNPs were recalculated for each sample due to changes in MAF across the population due to removal of strains. Phenotypes were then subset and GWAS run through fastLMM (Lippert et al. 2011) followed by analysis of how many significant SNPs were maintained after subsetting as well as how many SNPs which had been present in the original panel were lost due to shifts in MAF.

## Results

### Bacterial phylogenetic regression studies

**Fig. 2.**
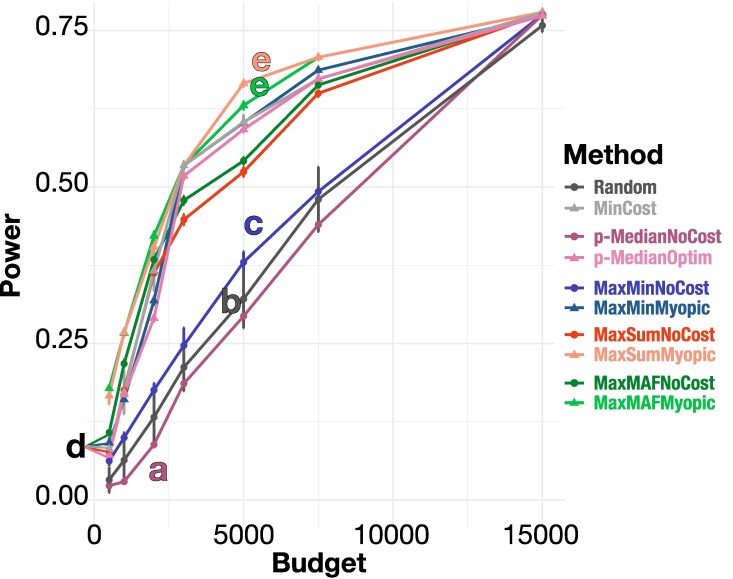
Power of phylogenetic regressions on traits simulated across 165 commercially available Lachnospiraceae isolate genomes. Methods are colored by how they consider genetic diversity, with lighter colors corresponding to methods that consider cost (MinCost and the Myopic and Optim methods). Error bars are ±1 SD of the mean of 20 replicates. Lowercase letters (a to d) indicate significantly different groups; methods are significantly different at *P* = 0.05 if they do not share a letter (GAM beta regression, Tukey-adjusted post hoc pairwise tests; see Methods).

We considered 2 scenarios for our test of strain selection methods on a bacterial dataset. In the first of these, we used the Lachnospiraceae phylogenetic tree and genotypes that we inferred from the genomes, with genotype represented as the presence or absence of “phylogroups” (sets of orthologs that had identical phylogenetic profiles). We then simulated phenotypes correlated with a randomly selected phylogroup, such that a phylogenetic regression on the full panel would have approximately 80% power to detect an effect at a nominal *P*-value ≤ 0.05 (Fig. 2, Supplementary Fig. 2).

Selecting strains randomly, as expected (Random), fared poorly in terms of power. More interestingly, though, despite the amount of prior literature focusing on these methods, we found that the greedy MaxMin method that did not consider costs was barely better than random guessing, and the heuristic for the p-Median problem without costs was actually even worse. We believe this poor performance can be explained by the fact that costs in our panel varied over more than an order of magnitude (from $37.33 to $474.00), and so methods that did not consider costs explicitly tended to select far fewer strains than their cost-aware counterparts (Table 1).

**Table 1.** Median number of Lachnospiraceae strains selected and range (in brackets), by method and budget (“Real” simulation).

Budget	500	1,000	2,000	3,000	5,000	7,500
Random	4.6 [1–7]	10.4 [6–14]	22 [15–28]	29.2 [23–36]	54.8 [40–62]	74.8 [64–86]
p-MedianNoCost	5 [5–5]	6 [6–6]	20 [20–20]	34 [34–34]	54 [54–54]	84 [84–84]
MaxMinNoCost	8.2 [7–9]	14 [14–14]	27.6 [26–28]	36.2 [33–40]	64.8 [60–68]	90.2 [89–92]
MaxSumNoCost	9 [9–9]	14.4 [14–16]	26.4 [26–28]	39.2 [38–40]	53.8 [53–55]	94 [94–94]
MaxMAFNoCost	9 [9–9]	14 [14–14]	26 [26–26]	40 [40–40]	61 [61–61]	93 [93–93]
p-MedianOptim	13 [13–13]	26 [26–26]	51 [51–51]	78 [78–78]	97 [97–97]	119 [119–119]
MaxMinMyopic	13 [13–13]	26 [26–26]	53 [53–53]	79 [79–79]	97 [97–97]	119 [119–119]
MaxSumMyopic	13 [13–13]	26 [26–26]	53 [53–53]	79 [79–79]	97 [97–97]	119 [119–119]
MaxMAFMyopic	13 [13–13]	26 [26–26]	53 [53–53]	79 [79–79]	98 [98–98]	119 [119–119]
MinCost	13 [13–13]	26 [26–26]	53 [53–53]	79 [79–79]	98 [98–98]	119 [119–119]

Even the cost-aware versions of MaxMin or p-Median, however, were not the strongest performers overall. In fact, even the non-cost-aware MaxSum method, which maximizes the total sum of pairwise distances, and the MaxMAF method, which maximizes the total sum of MAFs, had performance that approached the cost-weighted version of MaxMin and p-Median. The cost-aware versions of MaxMAF and MaxSum preserved even more power and, indeed, were essentially tied for the highest performance overall. This difference was especially pronounced at lower budgets (e.g. $500 to $1,000).

We were surprised to see that one of the simplest methods—picking strains in order of cost, without considering diversity at all (MinCost)—was the third-ranked, behind MaxMAFMyopic and MaxSumMyopic, and was not significantly worse than these across the range of budgets. MinCost even beat the globally optimized p-MedianOptim method in these simulations (though not significantly). As above, a possible explanation is that MinCost always maximizes the number of strains selected (Table 1), which, all else being equal, should always increase power.

**Fig. 3.**
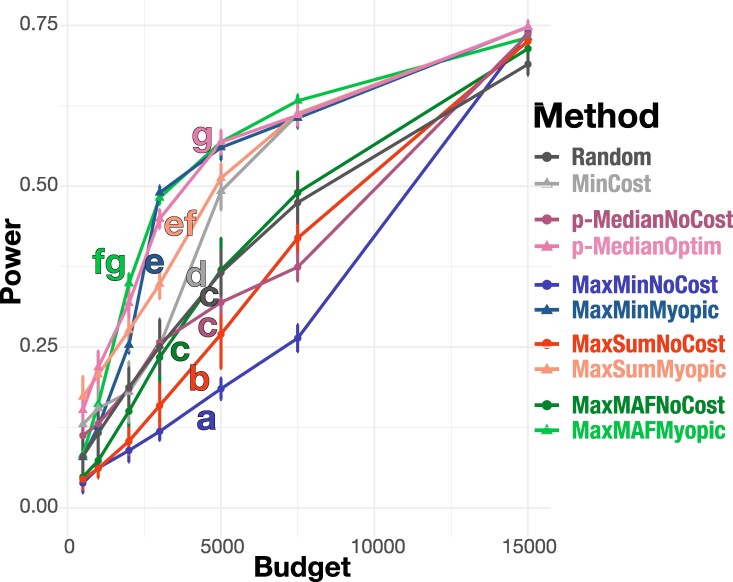
Power of phylogenetic regressions on traits simulated across 165 commercially available Lachnospiraceae isolate genomes, augmented by 100 highly similar synthetic “oversampled” strains. Methods are colored as in Fig. 2. Error bars are ±1 SD of the mean of 20 replicates. Lowercase letters (a to d) indicate significantly different groups; methods are significantly different at *P* = 0.05 if they do not share a letter (GAM beta regression, Tukey-adjusted post hoc pairwise tests; see Methods).

We reasoned that we might expect to see MinCost perform worse in settings where the panel contained clusters of inexpensive but closely related strains, as these would make only marginal contributions to the diversity metrics we consider. In some collections, for example, certain individual species with high value in the wet-lab or industry, such as *Escherichia coli* or *Bacillus subtilis*, have been “oversampled” relative to more distant phylogenetic neighbors. We would not have expected to observe this phenomenon in the Lachnospiraceae dataset we considered above because this set does not contain common model or industrial microbes (at least, not yet). To test this hypothesis, we therefore simulated the addition of another collection that had a large number of inexpensive, closely related strains, which we refer to as “oversampled” strains below (Fig. 3, Supplementary Fig. 3).

In this “oversampled” scenario, we indeed saw a much larger advantage for the methods that consider both cost and diversity and also saw more differentiation among these methods. Strikingly, MaxMAFMyopic still performed the best overall, significantly outperforming MinCost across all budgets below the very highest (where all methods performed well). In this setting, the p-MedianOptim method was second-best overall. Because p-MedianOptim minimizes the distance of non-selected to selected strains, it should pick strains that are evenly distributed in terms of their genetic relatedness (see Fig. 1), which may explain why it is the most robust to oversampling a particular taxon. The next best methods were, in order, MaxSumMyopic and MaxMinMyopic. All 4 of these methods significantly outperformed MinCost across the range of budgets, again matching the intuition that prioritizing diversity becomes more important when a low-diversity clade is oversampled.

Unexpectedly, MaxSumNoCost and MaxMinNoCost were significantly worse than random guessing. Because methods that considered diversity in this experiment tended to perform so well, this was initially surprising. However, in this experiment, the highly similar strains we simulated were also very low cost ($20.00). This gives the Random approach an advantage, as it would tend to run out of its budget slower than the other NoCost methods, which are required to maximize diversity. Indeed, MaxMinNoCost and MaxSumNoCost picked the fewest total genomes out of any method (Table 2). We found there was no consistent relationship between performance and the number of synthetic “oversampled” strains that were selected (Table 3). MaxMinNoCost and MaxMAFNoCost picked the least but performed worst, while at low budgets (≤$5,000), p-MedianOptim selected more than Random and MaxMinMyopic.

**Table 2.** Median number of total strains selected and range (in brackets), by method and budget (“oversampled” simulation).

Budget	500	1,000	2,000	3,000	5,000	7,500
Random	8.8 [5–11]	12.4 [10–18]	27.2 [24–36]	38.4 [28–47]	74 [67–82]	104.8 [85–121]
p-MedianNoCost	8 [8–8]	15 [15–15]	31 [31–31]	41 [41–41]	59 [59–59]	74 [74–74]
MaxMinNoCost	8.6 [7–9]	14 [14–14]	22.8 [21–24]	28 [28–28]	48.4 [47–52]	65.2 [64–66]
MaxSumNoCost	7 [7–7]	12.4 [12–14]	19 [19–19]	30 [28–32]	44.6 [43–45]	74 [74–74]
MaxMAFNoCost	7 [7–7]	12 [12–12]	26 [26–26]	38 [38–38]	63 [63–63]	93 [93–93]
p-MedianOptim	16 [16–16]	30.4 [30–31]	58 [58–58]	85 [85–85]	117 [117–117]	141 [141–141]
MaxMinOptim	14 [14–14]	27 [27–27]	54 [54–54]	78 [78–78]	97 [97–97]	119 [119–119]
MaxSumMyopic	17 [17–17]	34 [34–34]	68 [68–68]	100 [100–100]	165 [165–165]	198 [198–198]
MaxMAFMyopic	14 [14–14]	27 [27–27]	54 [54–54]	81 [81–81]	102 [102–102]	121 [121–121]
MinCost	20 [20–20]	40 [40–40]	80 [80–80]	113 [113–113]	166 [166–166]	198 [198–198]

**Table 3.** Median number of the additional oversampled strains that were selected and range (in brackets), by method and budget (“oversampled” simulation).

Budget	500	1,000	2,000	3,000	5,000	7,500
Random	4.6 [2–7]	5.4 [3–9]	11.4 [8–13]	13.2 [8–17]	27.2 [23–31]	39.2 [28–49]
p-MedianNoCost	5 [5–5]	5 [5–5]	7 [7–7]	8 [8–8]	10 [10–10]	10 [10–10]
MaxMinNoCost	1.2 [1–2]	1.6 [1–2]	1.4 [1–2]	2 [2–2]	1.8 [1–2]	2 [2–2]
MaxSumNoCost	1.2 [1–2]	1.4 [1–2]	2 [2–2]	2.2 [2–3]	1.8 [1–2]	2 [2–2]
MaxMAFNoCost	2 [2–2]	2 [2–2]	4 [4–4]	5 [5–5]	10 [10–10]	16 [16–16]
p-MedianOptim	8 [8–8]	11.2 [10–13]	14 [14–14]	15 [15–15]	27 [27–27]	29 [29–29]
MaxMinMyopic	2 [2–2]	2 [2–2]	2 [2–2]	8 [8–8]	21 [21–21]	20 [20–20]
MaxSumMyopic	12 [12–12]	23 [23–23]	44 [44–44]	61 [61–61]	95 [95–95]	100 [100–100]
MaxMAFMyopic	2 [2–2]	1 [1–1]	2 [2–2]	2 [2–2]	5 [5–5]	2 [2–2]
MinCost	20 [20–20]	40 [40–40]	80 [80–80]	100 [100–100]	100 [100–100]	100 [100–100]

Overall, in these isolate studies, failing to consider costs appeared to harm performance more than failing to consider diversity. In fact, in the presence of strongly unequal costs, maximizing diversity alone was sometimes worse than random guessing. However, the best methods—namely, the cost-aware versions of MaxSum, MaxMAF, and, especially in the presence of oversampled clades, p-Median—did consider both.

### Mouse genome-wide association studies

We next evaluated these methods using simulations conducted in a substantially different context: genome-wide association studies (GWAS) on subsets of the HMDP, which consists of approximately 175 strains of mice. At our selected effect size (χ^2^) and MAF thresholds, we observed approximately 50% power to detect a true positive result in ∼60 strains at a χ^2^ of 100 and a MAF of 10% to 30% or 100–110 strains at a χ^2^ of 50 at various MAF thresholds (Supplementary Fig. 1a). At χ^2^ = 100, there was 100% power to detect phenotypes in the whole panel, while at χ^2^ = 50, there was 82% to 93% power to detect a phenotype.

**Fig. 4.**
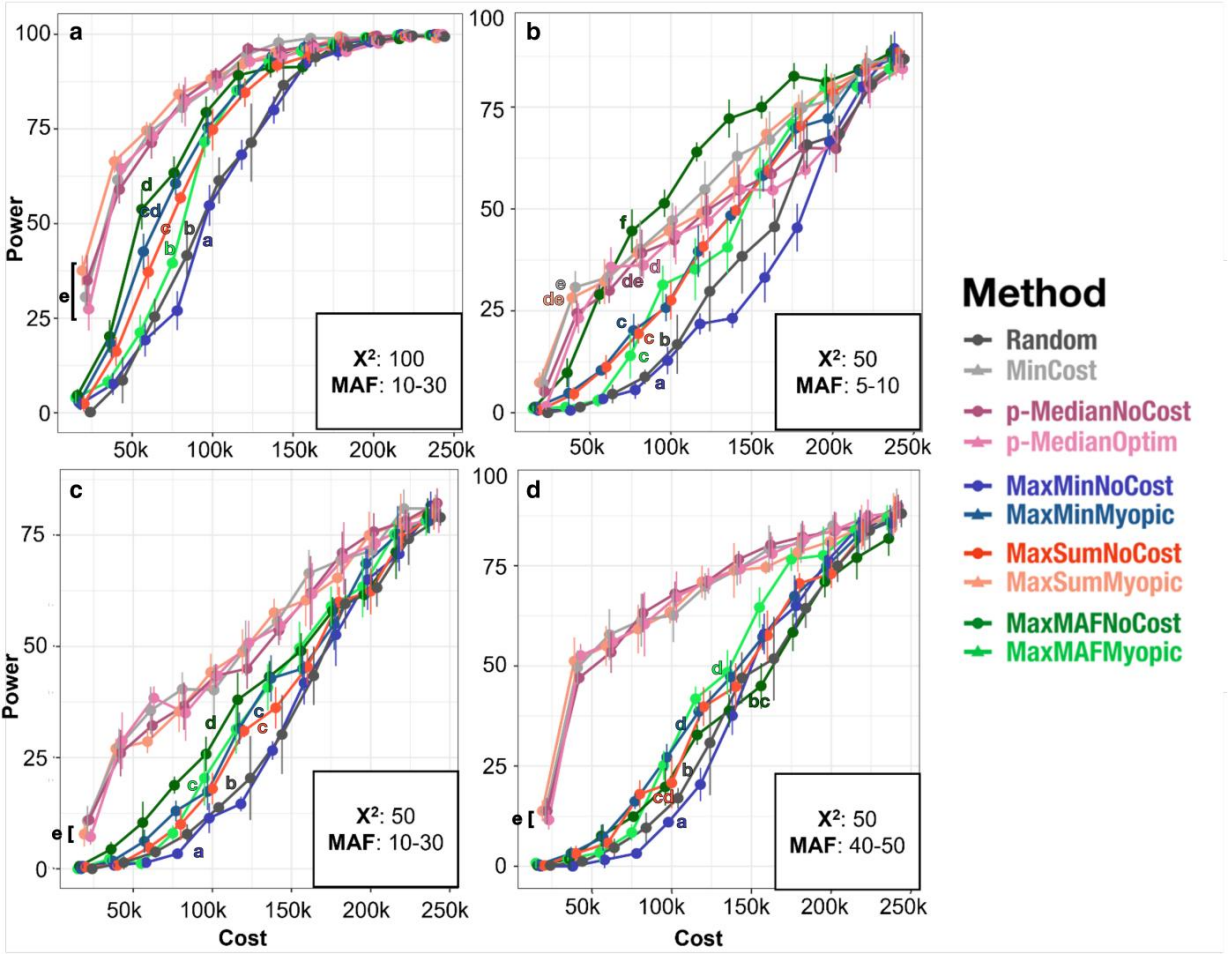
Mouse GWAS power simulations without additional costs per strain. Results of simulation studies at $20k intervals from 20 to 240 k. a) χ^2^ of 100 and MAF of 10% to 30%, b) χ^2^ of 50 and MAF of 5% to 10%, c) χ^2^ of 50 and MAF of 10% to 30%, d) χ^2^ of 50 and MAF of 40% to 50%. *N* = 100 simulated phenotypes for each of 5 replications. Values are slightly jittered on the *x* axis to allow for observation of each method's error. Error bars are ±1 SD. Lowercase letters (a to f) indicate significantly different groups; methods are significantly different within a panel at *P* = 0.05 if they do not share a letter (GAM beta regression, Tukey-adjusted post hoc pairwise tests; see Methods).

With this understanding of how random strain selection is likely to affect GWAS results in our panel, we set out to examine the behavior of each of selection approaches at price limits ranging from $20k to $240k (approximately the cost of the entire panel). Across all χ^2^ and MAF parameters (Fig. 4), we saw a very rapid jump in power in some models at low cost thresholds (see, for example, the jump from 10% to 50% power in Fig. 4d from $20k to $40k). This reflects the specific cost structure of these strains: there are a large number of relatively inexpensive mice that can all be obtained at $40k total budget, while many of strains found in the Recombinant Inbred panels are significantly more expensive.

Several results line up with our observations on the bacterial lines. The Random, MaxSumNoCost, and MaxMinNoCost algorithms all performed significantly worse (Tukey-adjusted p < 0.05) than their cost-aware Myopic counterparts. Typically, MinCost also remained in a statistical dead heat for the top rank.

However, other methods showed reversals from our bacterial simulations. We observed that MaxMinNoCost, instead of p-MedianNoCost, was either the worst or second-worst performer at all cost/χ^2^/MAF combinations, and in fact, p-MedianNoCost was actually tied for the top place. Most surprisingly, MaxMAFMyopic, previously in the top 2 methods, performed worse than average. In fact, MaxMAFMyopic was often beaten significantly not only by MaxSumNoCost but by its own cost-unaware counterpart, MaxMAFNoCost (Fig. 4).

These differences did not depend on effect size, which mainly affected how much power could be retained overall (compare Fig. 4a and 4c). As expected, for very strong signals (χ^2^ = 100; Fig. 4a), power rose faster with the available budget, with the best methods reaching 90% power at a $100 K budget, compared to less than 50% for weaker signals (χ^2^ = 50; Fig. 4c). However, in both cases, methods clustered into approximately the same 3 groups: the best were MaxSumMyopic, p-MedianOptim, p-MedianNoCost, and MinCost; a second tranche included both versions of MaxMAF, MaxMinMyopic, and MaxSumMyopic; and the worst were Random and MaxMinNoCost.

In contrast, certain methods did show MAF-dependent behavior. We assessed this by sampling 3 different MAF cutoffs at an effect size of χ^2^ = 50: 5% to 10%, 10% to 30%, and 40% to 50% (Fig. 4b–d). We hypothesized that for low MAFs, methods that prioritize rare variants should maximize power. This is because low MAFs mean that the causal SNPs are rare, and because χ^2^ was held constant, it then follows that individual rare SNPs must have larger effects on the phenotype.

Indeed, we observed that MaxMAFNoCost, which would favor adding strains with rare variants, was the method affected most dramatically by allele frequency. When causal SNPs already have MAFs close to 50%, prioritizing minor allele recovery should be unnecessary, and because it does not consider costs, MaxMAFNoCost would then lose power by selecting fewer strains (Supplementary Fig. 1b). Sure enough, at MAFs of 40% to 50%, MaxMAFNoCost performed poorly, at times worse than random selection (Fig. 4d). At moderate MAF (10% to 30%), MaxMAFNoCost and MaxMAFMyopic performed similarly and in the middle of the set of models in terms of power recovery: comfortably above Random but significantly below MinCost (Fig. 4c). At low MAFs (5% to 10%), however, MaxMAFNoCost actually became the best model for higher budgets, at times recovering 10% more power than the second-best model (Fig. 4b). This is likely because rare SNPs carry most of the signal in the data, and so while MaxMAFNoCost picks fewer strains (a bigger problem with very low budgets), it also tends to pick the “right” strains for recovering the signal.

While we have previously only considered the cost of acquiring the strains, in most real-world scenarios, downstream phenotyping experiments require additional costs that scale per animal or strain, placing further economic constraints on how many strains it is feasible to purchase. To simulate these additional costs (which could include RNAseq, histology, experimental manipulation, etc.), we added $2,500 to the cost of each mouse strain. This also smooths out the very large cost differences seen in the mouse data, such that now the most expensive strain would only be 2× that of the least expensive strain, rather than >40×. Indeed, we see smaller differences in the number of mouse strains chosen between the models (Supplementary Fig. 1c).

**Fig. 5.**
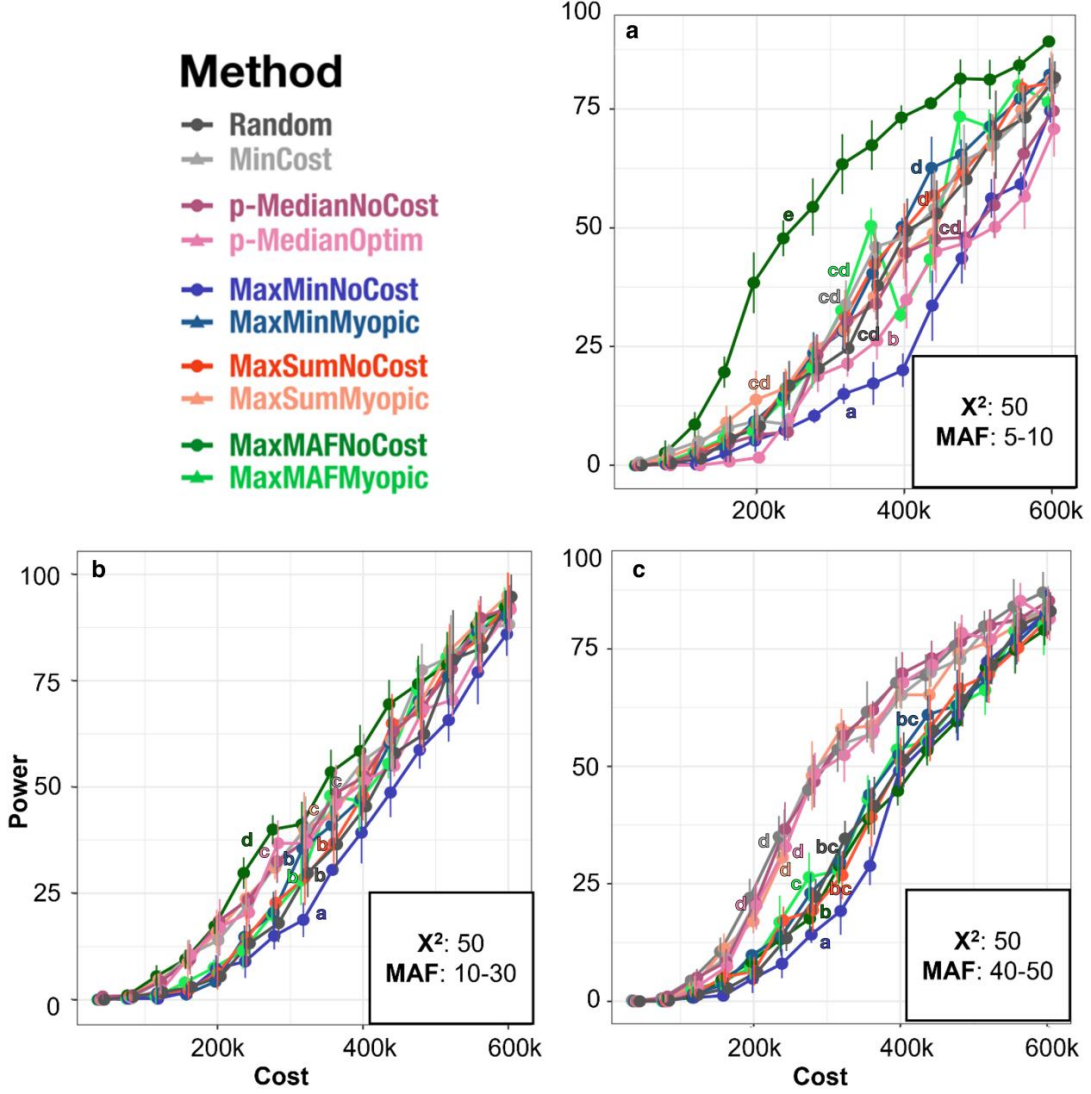
Mouse GWAS power simulations with additional cost per strain. Results of simulation studies after $2,500 was added to the cost of each strain at $40k intervals from 40 to 600k. a) χ^2^ of 50 and MAF of 5% to 10%, b) χ^2^ of 50 and MAF of 10% to30%, c) χ^2^ of 50 and MAF of 40% to 50%. *N* = 100 simulated phenotypes for each of 5 replications. Values are slightly jittered on the *x* axis to allow for observation of each method's error. Lowercase letters (a to f) indicate significantly different groups; methods are significantly different within a panel at *P* = 0.05 if they do not share a letter (GAM beta regression, Tukey-adjusted post hoc pairwise tests; see Methods).

With a flatter cost structure, MaxMAFNoCost's advantage on low MAF SNPs widened even more across the entire budget range (Fig. 5a), at times beating the next best model by 25%. Furthermore, it became the top-ranked method at moderate MAFs as well (Fig. 5b). As we discussed above, MaxMAFNoCost is more likely to pick more “useful” strains but also fewer, leading to a power tradeoff. When strain costs become more similar, this second factor should become less important, which explains the improvement in power across MAF thresholds. Crucially, however, MaxMAFNoCost still performed poorly when MAFs were between 40% and 50% (Fig. 5c). Thus, maximizing MAF without regard to cost is a viable strategy when causal alleles have low frequency and especially as cost differentials decrease, but not when MAFs are already close to parity.

With less differentiation by cost, the other competing methods became more similar to one another (Fig. 5a and b), except for MaxMinNoCost, which remained weaker than random guessing. We also did not observe the same early jump in power we saw previously, which was expected as there was less of a cost differential between the cheapest and most expensive strains. Interestingly, MaxMAFMyopic's performance became more variable, especially at low MAF, but still lagged behind the others. MinCost, in contrast, remained statistically tied for the best or second-best method across these simulations.

Counterintuitively, optimizing for either cost (MinCost) or total MAF (MaxMAFNoCost) separately was effective for picking mouse strains, optimizing for both (MaxMAFMyopic) did not appear to be, even though it had been the best approach for bacteria. We entertained 2 explanations for this result. First, there could be a bias where more expensive mouse strains contained rarer alleles, meaning that the strains that maximize MAF were intrinsically high-cost. Second, it could be that the greedy MaxMAFMyopic algorithm simply did not get close enough to a global optimum on the mouse data. This failure could be due to differences in the genetic architecture of the 2 groups: the mouse genomes include recombinant inbred lines (RILs), while the bacteria are exclusively natural isolates from many different species.

We tested the first of these hypotheses by randomly distributing costs (pulling from a uniform distribution from $60 to $1,000 per strain) and re-running the simulations. When we did so, we still observed that MaxMAFNoCost preserves more power than MaxMAFMyopic (Tukey-corrected *P* < 0.05, Supplementary Fig. 4), indicating that a correlation between cost and rare alleles does not drive this pattern. To test the second explanation, we took a set of bacterial genomes and generated simulated “recombinant” strains, whose genotype at each locus was randomly sampled from the “parental” strains, and then repeated the analysis. We found that in this experiment, all of the cost-aware approaches performed similarly, outperforming the non-cost-aware methods, with the sole exception of MaxMAFMyopic (Supplementary Fig. 5). Together, these 2 results favor the explanation that a simple myopic method may not effectively co-optimize MAF and cost when the full panel contains a large number of recombinant lines, which largely contain different permutations of the same variants, as opposed to isolates, which have more unique variants per strain.

In these experiments, MaxMAFNoCost had unusually high but also variable performance. We therefore conducted additional simulations exploring how its performance was affected by effect size (χ^2^), MAF, and budget (Supplementary Fig. 6). These simulations used the mouse panel with additional costs, as this was the scenario where MaxMAFNoCost performed best (compare, e.g. Figs. 4 and 5). We tested MaxMAFNoCost against the simplest cost-aware method, MinCost, across 5 different χ^2^ values spanning a larger range than our previous experiments (20–100), as well as across 9 more fine-grained MAF thresholds from very rare to very common (5% to 50%). We found that for every χ^2^ value, MinCost performed better when the MAF exceeded 15%, while for lower MAFs, MaxMAFNoCost was more powerful. Furthermore, MaxMAFNoCost's advantage was most pronounced at lower effect sizes. In the worst cases, the difference in power between MaxMAFNoCost and MinCost could exceed 2-fold, indicating that these methods sacrificed substantial power on either common or rare alleles, respectively. Together, these results suggest that MaxMAFNoCost has specific utility in situations where the recovery of every minor allele is crucial *and* the cost structure of the strains is relatively flat, meaning that MinCost cannot boost its power purely by having a much higher sample size.

### GWAS validation in the HMDP

Finally, we applied our selection criteria to real data pulled from a prior HMDP study on cardiac dysfunction (Rau et al. 2015b). In particular, we examined the effects of strain selection on GWAS loci for right ventricle weight after catecholamine challenge, which had the largest number of genome-wide significant SNPs in the original study (Fig. 3a).

**Fig. 6.**
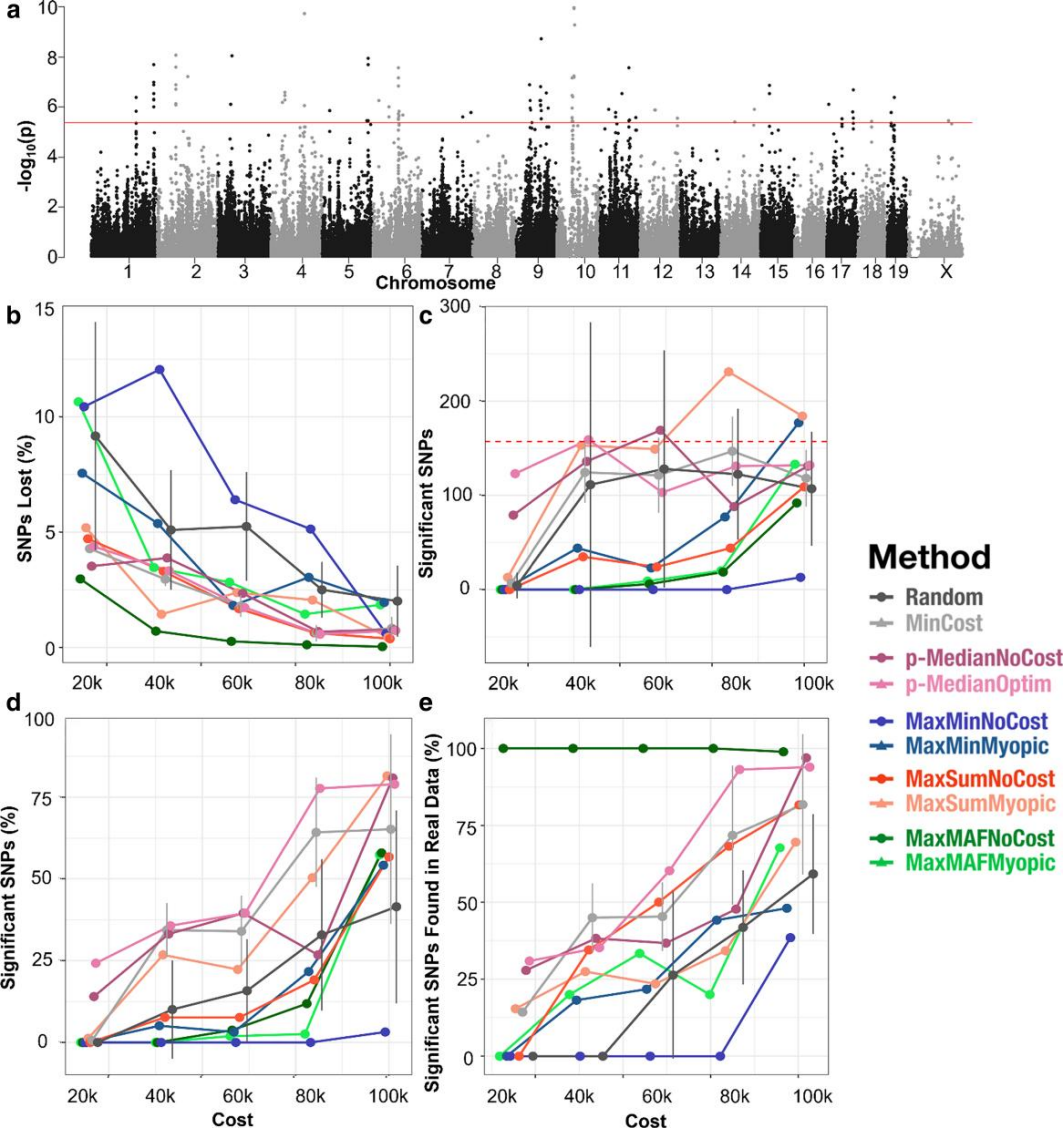
Mouse GWAS on actual data. Results of subsetting studies (*N* = 10 permutations each) for real data pulled from the HMDP Heart Failure Study(Rau et al. 2015b) at $20k cost intervals from 20 to 100k. a) Manhattan plot of the results of the whole panel; the horizontal line represents the genome-wide significance threshold of a FWER of 5% (Bennett et al. 2010). b) The percentage of SNPs untested in the subsets because of changing MAFs. c) The number of significant SNPs found by each approach; the dotted horizontal line indicates the number of significant SNPs found in the whole panel. d) The percentage of preserved genome-wide significant SNPs after subsetting between the subset and the full panel. e) The percentage of found significant SNPs which are also significant in the whole panel GWAS. Values are slightly jittered on the *x* axis to allow for observation of each method's error.

To be consistent with the original study, SNPs with a MAF of less than 5% were excluded; because strain selection affects this calculation, we first examined the effects of removing strains on the number of SNPs tested. As expected from our simulations, MaxMinNoCost and Random resulted in the largest number of SNPs being lost, over 10% at $20 K and 5% at $60 K. MaxMAFNoCost predictably lost the fewest number of SNPs, <3% at $20 K and only 0.3% at 60 K (Fig. 6b). MinCost, despite not considering genetic diversity at all, did reasonably well, with 4.3% SNP loss at 20 K and 1.8% loss at 60 K.

We next queried how many genome-wide significant SNPs were identified for each method at each cutoff when compared to the full panel (Fig. 6c). Strikingly, at $80 K and $100 K cutoffs, MaxSumMyopic actually identified more genome-wide significant loci than the full panel, joined by MaxMinMyopic at the highest threshold. Random selection resulted in very high variance, with some permutations returning as many as 433 significant SNPs (compared to 157 found in the real data), while others at the same $60 K threshold returned as few as 27. Consistent with our simulations, MaxMinNoCost recovered very few significant SNPs, returning zero significant hits at every budget other than the highest. We were surprised that the 2 MaxMAF algorithms also performed poorly; however, after investigating, we found that many of the significant SNPs in the full panel had MAFs >20%, a setting where MaxMAF performed less well in simulations (Fig. 4c and d, Supplementary Fig. 6).

Finally, we tested how well the results from our sub-panels agreed with those from the real panel. We first asked what the percentage of significant SNPs from the real panel were recovered in our sub-panels (Fig. 6d); assuming that the SNPs from the real panel are valid, this is a measure of recall. Here, p-MedianOptim and MinCost performed the best, recovering about 37% of all SNPs at $60 K and, in the case of p-MedianOptim, 75% at 80 K. Notably, despite returning the most overall SNPs, with comparable numbers to the full panel starting at $40 K and higher by $80 K, MaxSumMyopic did not recover a majority of the SNPs from the real panel until reaching $100 K, suggesting an elevated false-positive rate (FPR).

We next calculated a measure of precision: the percentage of SNPs recovered by the sub-panels that were also found in the real panel (Fig. 6e). We indeed found that MaxSumMyopic had fairly low precision. Conversely, MaxMAFNoCost, despite returning very few significant SNPs, was very faithful to the full panel, with 100% of its recovered SNPs being reproduced (other than at $100k, where it drops to 98%). Again, the best methods were p-MedianOptim, followed by MinCost, with rates between 45% and 80% starting at $40 K onward. Thus, MinCost and especially p-MedianOptim best optimized both precision and recall, while the selections made by MaxMAFNoCost strongly favored precision.

## Discussion

In this study, we set out to address a common problem faced by researchers: how best to select strains from an available panel in order to stay within a fixed budget while retaining the power needed to make reliable discoveries. Because such comparative approaches are used in many different fields of study within biology, we simulated experiments on phylogenetic regression in microbes and on GWAS in mice, followed by subsampling of real mouse GWAS data as a validation.

### Cost-awareness is at least as important as diversity optimization

Across both bacterial phylogenetic regression and mouse GWAS, the most consistent—and surprising—finding was that explicitly accounting for strain cost mattered as much as, or more than, accounting for genetic diversity. This is notable, given that the large majority of strain selection algorithms explicitly focus on the latter while ignoring the former (Pardi and Goldman 2005; Steel 2005; Kang and Marjoram 2012; Zhang et al. 2013). Weitzman's original “Noah's Ark” formulation of the problem based on phylogenetic diversity (Faith 1992) did incorporate costs (Weitzman 1998); Hartmann and Steel (Hartmann and Steel 2006) and later Pardi and Goldman (Pardi and Goldman 2007) also developed efficient algorithms for maximizing phylogenetic diversity under cost constraints. However, empirical analyses of how real-world cost differences impact strain selection and, especially, power in downstream genetic association studies, have been lacking. Our results provide clear evidence that when strain prices vary substantially (over an order of magnitude), methods that ignore costs tend to select far fewer strains: this results in lower sample sizes and therefore much lower power. In most scenarios, this disadvantage overwhelms any advantage from considering diversity.

The surprisingly strong performance of MinCost, in which the algorithm simply selects the cheapest strains until reaching the budget threshold, is an illustrative case. Why does a method that does not consider diversity perform so well? One explanation is that the panels we used were already themselves very diverse. Researchers who contribute to these collections are likely motivated, at least in part, by considerations like expanding the diversity of cultured microbes to which we have access, or in the case of mice, breeding lines that aim to randomly shuffle genotypes through recombination. In the context of a panel that is already diverse, “buy as many as you can afford” becomes a viable strategy because it maximizes sample size, echoing a general principle of quantitative genetics that sample size is often the dominant determinant of statistical power.

However, our results also show that it is often still possible to beat MinCost. This is likely because power depends not just on total sample size but also specifically sampling informative genotype/phenotype pairings. In the extreme, if only one allele were ever detected at a locus, it would be impossible to associate it with a phenotype. Typically, larger sample sizes translate into more observations of minor and major alleles, and therefore to more variation at each locus, but this may not always be true: for example, when causal alleles are rare (Fig. 4, Supplementary Fig. 6) or when many of the strains come from a single taxon (Fig. 3). MinCost struggles in these situations. Additionally, when cost differences are not as pronounced, there may be many subsets that approximately maximize the sample size for a given budget; MinCost can only choose randomly between these, eroding its main advantage (Fig. 5). In the above specific scenarios, we found that methods considering both cost *and* diversity—in particular p-MedianOptim, MaxMAFMyopic, and MaxSumMyopic—were more effective. This suggests that MinCost is a safe default approach when a panel's diversity is well-distributed across the price range but may not be appropriate in situations where prices are not as differentiated or where sample size is a weaker proxy for the amount of variation at causal loci.

### MaxMin methods are outperformed by alternative approaches

Despite being heavily studied for strain selection (Kuby 1987; Hartmann and Steel 2006; Bordewich et al. 2008), MaxMin was consistently among the weakest performers in our study. This is an important observation, as in cost-unaware situations, greedy MaxMin algorithms have been widely recommended: they have been shown to perform well in bioconservation contexts (Bordewich et al. 2008) and, under certain assumptions (e.g. distance on ultrametric trees), also maximize phylogenetic diversity (Pardi and Goldman 2005; Steel 2005). However, maximizing power in a genetic association study is a different goal than maximizing diversity for bioconservation and may therefore require a different approach. Indeed, McAuliffe and colleagues (McAuliffe et al. 2005) showed that the taxa selected to maximize power in a comparative genomics study often substantially differ from those that would maximize their evolutionary divergence. Our results echo this finding and expand on it by showing that it also matters how exactly diversity is maximized, as certain approaches result in much higher power than others.

MaxSum, for example, consistently outperformed MaxMin. One reason for MaxSum's superior performance may be that MaxMin maximizes the distance to the nearest selected strain. In contrast, the MaxSum method maximizes the sum of all pairwise distances, meaning that being closely related to one already-selected strain can be balanced out by being very diverged from most others. Thus, MaxSum may be less likely to reject potentially informative strains that would be deprioritized by MaxMin. However, it is also important to note that in our tests on real data, MaxSum did appear to lead to an elevated FPR. The reason for this is not yet clear. When we assessed the FPR in our bacterial simulations from Figs. 2 and 3, we found some variability across methods at high MAFs and low budgets, but MaxSum's FPR was not unusually high (Supplementary Fig. 7). We also investigated whether using MaxSum might distort the population structure (Rau et al. 2015a) or whether it might tend to pick strains with more extreme phenotypes; in preliminary tests, however, neither explanation was supported. Therefore, despite its promising performance in simulations, we would recommend caution in applying MaxSum until its behavior on real-world experimental data is better understood.

MaxMAF showed even stronger performances in some settings, consistent with the logic of the “M Strategy” from crop science that aims to maximize allelic diversity in subsets and has been showed to retain fewer non-informative alleles than divergence-based approaches (Schoen and Brown 1993; Franco et al. 2006; Kim et al. 2007). In the context of association studies, this push toward MAFs of 0.5 maximizes the power of the binomial-based regression test at the heart of the association study, meaning that pushing allele frequencies toward 50% directly promotes the conditions under which gene–phenotype associations are most detectable. Also, unlike MaxSum, MaxMAF did not lead to more false positives on real data.

Finally, p-MedianOptim performed the best on the real HMDP cardiovascular GWAS data (Rau et al. 2015b), providing the best balance of true to false positives for recovering known GWAS loci. The p-Median approach, by minimizing average distance from all strains to the nearest selected representative strain (Faith 1994; Matsen et al. 2013; Kang et al. 2015), selects subsets that are broadly representative of the full panel's genetic structure, which likely contributes to its performance. The p-MedianOptim method was also competitive with MinCost in most other scenarios and significantly outcompeted it in the presence of oversampled taxa.

### Genetic architecture and allele frequency modulate method performance

The 2 settings we tested represent different types of genetic diversity: cross-species variation in bacterial gene family content and within-species variation in mouse genetic polymorphisms. The bacterial genomes show strong phylogenetic nesting and generally low MAFs, while the mouse panel contains RILs specifically bred to tease apart otherwise correlated genetic variants (Ghazalpour et al. 2012; Davis et al. 2013; Lusis et al. 2016; Cao et al. 2022; Lahue et al. 2025). These architectural differences help explain several setting-specific results.

Most notably, MaxMAFMyopic performed well in bacteria but poorly in mice, a result that could not be explained by changing the cost structure (Supplementary Fig. 4), but could be reproduced when we generated simulated recombinant bacterial strains (Supplementary Fig. 5). Since the no-cost version of MaxMAF actually performed well, this suggests that the greedy heuristic may fail to effectively co-optimize MAF and cost when the panel contains many recombinant lines that share permutations of the same variants. Of course, greedy optimization provides no guarantees of reaching a global optimum. Better heuristics and more efficient strategies (Erkut et al. 1994) may resolve these limitations for MaxMAF and may also improve on some of our other myopic approaches.

Allele frequency had a strong effect on MaxMAFNoCost's performance: it excelled when causal alleles were rare (MAF 5% to 10%) but was poor when they were common (MAF 40% to 50%). As noted above, this appears to be due to an inherent tradeoff between preserving overall sample size and preserving the number of observations of a particular rare allele (Supplementary Figs. 2, 3 and 6). This is especially true at low effect sizes, which can be explained by noting that a large effect size may allow a particular association to be detected with only a few examples, while weaker effect sizes require more observations. In contrast, when most causal variants are common, the overall sample size becomes more important, which the NoCost methods are worse at preserving. As we observed, flattening the cost structure of the panel would attenuate this sample-size penalty, explaining why MaxMAFNoCost improves in performance. Overall, these results are also consistent with MaxMAFNoCost's performance on our real dataset, where most of the “true” SNPs (i.e. those that were significant in the whole panel) had at least moderate MAFs (>20%). In this setting, MaxMAFNoCost showed near-perfect precision but very low recall.

Consequently, MaxMAFNoCost may be well-suited for the very specific situation where the study population has a flat cost structure, the key alleles are expected to have a very low (5–10%) frequency, and the effect size per allele is expected to be small. At higher MAFs (>15%), regardless of the effect size, MinCost or other cost-aware approaches would tend to perform better.

### Overall recommendations

**Fig. 7.**
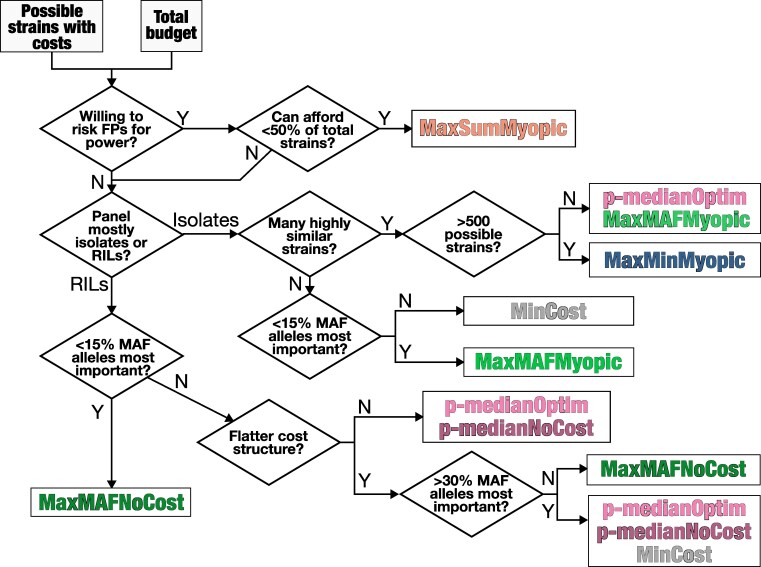
Flowchart for method selection.

Which method, then, is the best? As we have demonstrated, this is not a simple question to answer, but based on our results, we can make certain general recommendations. MinCost is one of the best methods overall. It is conceptually simple, is computationally tractable, and does not require detailed knowledge of strain genetics. However, it can fail when there are many cheap strains that are all related or when most causal alleles are expected to be rare and to have lower individual effect sizes. The p-MedianOptim approach is more computationally demanding and may not be tractable for very large panels. However, it is also robust to genetic structure, performed the best when applied to the experimental mouse data, and was at least statistically tied with MinCost in most other scenarios. The MaxMAF objective is useful when causal SNPs have lower MAFs and effect sizes but is more challenging to effectively co-optimize with cost, especially when the panel contains a large number of recombinant strains. Finally, MaxSumMyopic had very high power in simulations but showed excess false positives on real experimental data. To help guide researchers in determining the best approach for their situation, we have provided our full recommendations as a table (Table 4) and as a flowchart (Fig. 7).

**Table 4.** Recommended algorithms based on researcher's population of interest and expected results.

Model	Specific Use Cases	Specific Contraindications
Recommended		
p-MedianOptim	Many closely related, cheap strains OR recombinant inbred lines	Very large number of strains to choose from OR no closely related, cheap strains
MinCost	Very large number of strains to choose from MAF >15% alleles most important	Many closely related, cheap strains OR flat cost structure
Special Cases		
MaxMAFMyopic	Natural isolates AND Medium-to-large total budget	Very large number of strains to choose from OR recombinant inbred lines OR budget <25% of total
MaxMAFNoCost	Low MAF alleles are predicted drivers AND (lower per-allele effect sizes expected OR flat cost structure)	Very large number of strains to choose from MAF <15% alleles most important High per-allele effect sizes expected Unequal cost structure
p-MedianNoCost	Recombinant inbred lines AND MAF >15% alleles most important	Natural isolates OR (flat cost structure AND<30% MAF alleles most important)
MaxSumMyopic	Very large number of possible strains to choose from Willing to risk more false positives for high power Budget <50% of total	Not willing to risk higher false positives Very large total budget
MaxMinMyopic	Natural isolates AND Many closely related, cheap strains AND budget <50% of total	Recombinant inbred lines OR Small total budget
Not Recommended		
Random	—	Worse than MinCost
MaxSumNoCost	—	Worse than MinCost
MaxMinNoCost	—	Worse than Random

### Future directions

We primarily considered costs by using a version of Weizman's “myopic rule,” a greedy search that penalizes a strain's genetic distinctiveness by its cost. As noted, greedy searches are not guaranteed to reach a global optimum for every objective. We implemented global, cost-aware optimizers using MILP for p-Median, MaxMin, and MaxSum, but only the p-Median version proved to be computationally tractable on our panel sizes. That said, we used MILP formulations of MaxMin and MaxSum (Kuby 1987; Erkut 1990) that depended on “big-M” linearization, which may limit solver performance. More compact and efficient alternative formulations of these problems have been developed (He et al. 2010; Sayah and Irnich 2017; Lozano-Osorio et al. 2022), as well as new metaheuristic algorithms (Lozano-Osorio et al. 2022), which may allow them to be applied more broadly. Additionally, in this work, we used HiGHS, a modern and performant open-source solver, but it is also possible that proprietary solvers like Gurobi or CPLEX may have optimizations that would further improve performance. Thus, there is room to develop better, more scalable algorithms for balancing cost and diversity. We expect optimization efforts to be especially worthwhile in the case of MaxMAF, as our simulations indicate that MaxMAFMyopic's reduced performance on mouse data can be at least partly attributed to a failure of the simple greedy search to reach a global optimum.

In the current study, we modeled the effects of selecting subsets of strains from a larger cohort based purely on cost and/or genetic diversity. Other considerations, however, can also play a role in strain selection. For example, there is a wealth of previously generated data on a number of these strains available through websites such as the Mouse Phenome Database (Bogue et al. 2023) or BacDive (Schober et al. 2025) that may guide researchers to select certain strains with divergent phenotypes of interest as a “seed” around which to grow a larger panel (Bogue et al. 2023). This approach is compatible with the selection algorithms we described in this study, which can all accommodate a specific set of starting strains; integrating prior knowledge about phenotypic extremes may be a productive future direction for preserving power.

Finally, other future work could focus on better understanding the tradeoffs between cost, diversity, and minor allele coverage through multiobjective optimization (Dujardin and Chades 2018), understanding when and why applying the MaxSum method would result in false positives, and performing stability analyses across budgets and phenotypes.

While some power loss is unavoidable, our results do highlight that considering both cost and diversity may help mitigate this loss, allowing researchers to preserve more funding for downstream analyses or personnel costs. In particular, our results identify the MaxMAF and p-MedianOptim methods as especially promising and indicate that MaxSum also merits further study. At the same time, when the full panel is already diverse, our results also indicate that simply maximizing the number of purchased strains can be a surprisingly effective strategy.

## Supplementary Material

iyag137_Supplementary_Data

## Data Availability

Mouse Diversity Array genotypes are available from the Mouse Genome Informatics website (https://www.informatics.jax.org/). HMDP Phenotype data is available through Mendeley Resources at https://data.mendeley.com/datasets/y8tdm4s7nh/1. All code is available at https://github.com/pbradleylab/genome_annotation_matrix. Supplemental material available at GENETICS online.
