# Supplementary material for: Optimizing strain selection for association studies under hard cost constraints
Source: Genetics. 2026 May 27;233(4):iyag137. doi: 10.1093/genetics/iyag137 (PMC13439708; doi:10.1093/genetics/iyag137)
Supplement: Supplementary file 1 [file iyag137_supplementary_data.docx]

# Supplemental Figures

**Figure S1. Results of Random Strain Power Testing and Number of Strains Selected.** **A)** Results of 1,000 permutations at 5-strain intervals in the HMDP cohort for various χ^2^ and MAF thresholds **B)** Total number of strains selected when no additional costs are added per strain for each approach. **C)** Total number of strains selected when $2,500 is added to the cost of each strain to account for research costs **D)** Total number of strains selected for actual GWAS analysis. In all cases, x-values are shifted slightly to distinguish points, but budget cutoffs are the same as shown in Figures 4,5, and 6, respectively.

**Figure S2**. As in Figure 2 (reproduced in (A)), but stratified by MAF of the causal allele. MAF ranges are less than 0.1 (B), 0.3 ≤ MAF ≤ 0.5 (C), and 0.1 ≤ MAF ≤ 0.3 (D). Lowercase letters (a-g) indicate significantly different groups; methods are significantly different within a panel at p=0.05 if they do not share a letter (GAM beta regression, Tukey-adjusted post-hoc pairwise tests; see Methods).

**Figure S3**. As in Figure 3 (reproduced in (A)), but stratified by MAF of the causal allele. MAF ranges are less than 0.1 (B), 0.3 ≤ MAF ≤ 0.5 (C), and 0.1 ≤ MAF ≤ 0.3 (D). Lowercase letters (a-g) indicate significantly different groups; methods are significantly different within a panel at p=0.05 if they do not share a letter (GAM beta regression, Tukey-adjusted post-hoc pairwise tests; see Methods).

**Figure S4.** Power comparison between maxMAFNoCost and MaxMAFMyopic when costs per strain were randomly selected from a uniform distribution ranging from $60-$1000. Curves marked with “a” and “b” were significantly different in a post-hoc test of a beta-regression GAM at p=0.05 (see Methods). **A)** MAF 5-10%, **B)** MAF 10-30%, **C)** MAF 40-50%.

**Figure S5**. Power across budgets when “recombinant” bacterial strains were simulated from a set of six “parental” isolates. Error bars are ±1 SD across 50 replicates. MAF ranges are less than 0.1 (B), 0.1 ≤ MAF ≤ 0.3 (C), and 0.3 ≤ MAF ≤ 0.5 (D). Lowercase letters (a-c) indicate significantly different groups; methods are significantly different within a panel at p=0.05 if they do not share a letter (GAM beta regression, Tukey-adjusted post-hoc pairwise tests; see Methods).

**Figure S6**. Comparison of power across budgets between MaxMAFNoCost and MinCost at nine different MAF cutoffs. Panels A through E represent increasing allele effect sizes (χ^2^), as follows: A) 20, B) 35, C) 50, D) 75, and E) 100.

**Figure S7**. Estimated false positive rates for the “real” bacterial simulation shown in main-text Figure 2 (A-D) and the “augmented” simulations shown in main-text Figure 3 (E-H). Error bars are 1xSD from n=20 replicates. False positive rates are shown both overall (A, E) and stratified by MAF (B-D, F-H). MAF ranges are less than 0.1 (B, F), 0.1 ≤ MAF ≤ 0.3 (C, G), and 0.3 ≤ MAF ≤ 0.5 (D, H).
